# Prey-mediated effects of Mpp51Aa2-producing cotton on longevity and reproduction of *Orius majusculus*

**DOI:** 10.1007/s11248-024-00378-w

**Published:** 2024-04-05

**Authors:** Michael Meissle

**Affiliations:** Research Division Agroecology and Environment, Agroscope, Reckenholzstrasse 191, 8046 Zurich, Switzerland

**Keywords:** *Bacillus thuringiensis*, Environmental risk assessment, Genetically modified crops, *Gossypium hirsutum*, Natural enemies, Non-target effects

## Abstract

Genetically engineered (GE) cotton event MON 88702, producing Mpp51Aa2 (previously mCry51Aa2) from *Bacillus thuringiensis* (Bt), controls sucking pests, such as *Lygus* spp. (Hemiptera: Miridae) and thrips (Thysanoptera). Ingesting high doses of the insecticidal protein resulted in adverse effects on life table parameters of beneficial, predatory *Orius* spp. (Hemiptera: Anthocoridae). This triggered laboratory studies with more realistic food treatments, including different combinations of prey types with and without Bt protein to further characterize risks to this important group of non-target organisms. In this work, exclusive feeding of frozen spider mites (*Tetranychus urticae*, Acari: Tetranychidae) from Bt cotton confirmed adverse effects on longevity and fecundity of *O. majusculus* adults. Alternate feeding of Bt protein-containing spider mites and Bt-free *Ephestia kuehniella* (Lepidoptera: Pyralidae) eggs mitigated effects on longevity, but not on fecundity. When living larvae of *Spodoptera littoralis* (Lepidoptera: Noctuidae) from Bt cotton were fed to the predators, however, no effects on longevity and reproduction of female *O. majusculus* were observed, despite the fact that Bt protein concentrations in larvae were almost as high as concentrations in spider mites. When a diverse mix of prey species with various Bt protein concentrations is consumed in the field, it is unlikely that exposure of *Orius* spp. to Mpp51Aa2 is high enough to exert adverse effects on predator populations. MON 88702 cotton may thus be a valuable tool for integrated management of sucking pests.

**Supplementary Information:**

The online version contains supplementary material available at 10.1007/s11248-024-00378-w.

## Introduction

Biotechnology has been deployed to generate crop plants with resistance against major insect pests. Genetically engineered (GE) cotton event MON 88702 produces Mpp51Aa2 (previously mCry51Aa2), a modified insecticidal protein from *Bacillus thuringiensis* (Bt) (Gowda et al. 2016). While previous Bt cotton products carrying *cry1*, *cry2*, or *VIP* genes target Lepidoptera pests, MON 88702 effectively controls sucking pests, such as *Lygus* spp. (Hemiptera: Miridae) and thrips (Thysanoptera: Thripidae) (Akbar et al. 2019). In integrated pest management, the avoidance of broad-spectrum insecticides by using more environmentally friendly and selective pest control measures is key so that a rich complex of beneficial species can provide ecosystem services, such as biological control, decomposition, and pollination (Wijnands et al. 2012). Environmental risk assessment, which precedes every release of GE plants, includes an evaluation of potential non-target effects (Romeis et al. 2008). Because MON 88702 mainly targets bugs (Hemiptera, suborder Heteroptera), adverse effects on predatory bugs are of particular concern. In fact, when high doses of Mpp51Aa2 were provided in artificial diet, survival of 5-day-old *Orius insidiosus* (Say) (Heteroptera: Anthocoridae) nymphs was reduced (Bachman et al. 2017). In addition, spider mites from Bt cotton, which contained relatively high concentrations of Mpp51Aa2, caused impaired survival and development when fed to neonate *Orius majusculus* (Reuter) (Heteroptera: Anthocoridae) (Kim et al. 2021). Moreover, when 5-day-old nymphs were fed with spider mites from Bt cotton, the emerging females laid approximately 50% fewer eggs than females fed with spider mites from non-Bt cotton (Kim et al. 2021). In more realistic exposure scenarios with alternative prey that contained less or no Bt protein, such as noctuid larvae or cotton aphids, effects on the development of *O. majusculus* neonates were absent. When spider mites from Bt cotton were fed for limited time periods, effects on nymphs were less pronounced compared with exclusive Bt spider mite feeding (Boss et al. 2023).

The aim of this study was to further characterize potential risks of Mpp51Aa2-producing cotton on beneficial anthocorid bugs by assessing the effects of combinations of different prey types with and without Bt protein. While Boss et al. (2023) focused on juvenile development of *O. majusculus*, the current study investigated longevity and fecundity of adults. Exclusive feeding with spider mites or noctuid larvae reared on Bt cotton was compared with scenarios where prey from Bt cotton was alternated with flour moth eggs that contained no Bt protein.

## Material and methods

### Plants and insects

Cotton (event MON 88702 producing Mpp51Aa2, “Bt cotton”) and the non-transgenic near isoline DP393 (“non-Bt cotton”) were obtained from Bayer Crop Science (St. Louis, USA). Plants were cultivated in a climate chamber according to Boss et al. (2023) and used from 6 weeks after sowing. Mpp51Aa2 is the revised, structure-based name of modified Cry51Aa2 (Crickmore et al. 2021).

*Tetranychus urticae* Koch (Acari: Tetranychidae) spider mites were reared continuously on Bt or non-Bt cotton situated in one large environmental climate chamber (Boss et al. 2023). A preliminary experiment revealed that the number of spider mites needed for the planned experimental setup exceeded the capacity of the culture. Spider mites were thus collected over several months by beating the Bt or non-Bt cotton plants in the climate chamber over a tray and stored at − 70 °C. Instead of live prey, frozen spider mites were used for the here described experiments.

*Spodoptera littoralis* (Boisduval) (Lepidoptera: Noctuidae) was provided by Syngenta Crop Protection AG (Stein, Switzerland) and reared at Agroscope as described by Meissle et al. (2023). This species, also called cotton leafworm, is an Old World pest of cotton and thus a realistic prey of *O. majusculus*. Eggs of *S. littoralis* were collected daily. Every other day in the afternoon, neonates were placed on leaf discs from Bt or non-Bt cotton and incubated at 22 °C. In the next morning, living larvae were used for feeding experiments. The feeding time was kept short to ensure that larvae were still small (first instar) and thus easy to handle for *O. majusculus*. The green colour of the larvae and microscopic observations confirmed that their gut was filled with cotton tissue.

Alternative Bt protein-free prey consisted of *Ephestia kuehniella* Zeller (Lepidoptera: Pyralidae) eggs. While this storage pest is not a realistic prey for *O. majusculus*, it served as a model for Bt-free food that is known to have a high nutritional value for *O. majusculus* development and reproduction (Bonte et al. 2017; Boss et al. 2023). The species was not reared at Agroscope, but sterilized eggs were ordered from Agroline Bioprotect (Aesch, Switzerland), and stored at − 20 °C. No Bt-treatment was involved in the rearing of the species.

An *O. majusculus* culture was maintained on *E. kuehniella* eggs as food and bean pods as egg-laying substrate (Boss et al. 2023). To obtain specimens for experiments, batches of bean pods with fresh eggs (< 24 h) were incubated at 22 or 25 °C. Once eggs had hatched, bean pods were removed and nymphs were fed with *E. kuehniella* eggs until adult emergence.

### Feeding experiments with spider mites

Experiments started with adults that had emerged within the previous 48 h. After *O. majusculus* were sexed (Ferragut and González-Zamora 1994), one male and one female were introduced to a ventilated plastic dish (5 cm diameter), lined with a moist cotton cosmetic pad and a Bt or non-Bt cotton leaf disc (4 cm diameter). In the first series of experiments, the *O. majusculus* pairs were assigned randomly to one of five food treatments (Table 1): **SM:** prey consisted of frozen spider mites from either Bt or non-Bt cotton, presented on the Bt or non-Bt cotton leaf discs, respectively; **SM/EE alt.**: frozen spider mites from Bt or non-Bt cotton were fed alternately with frozen *E. kuehniella* eggs in 2-day periods, while half of the replicates started with mites as prey and the other half with eggs; **EE**: prey consisted of *E. kuehniella* eggs, presented on either Bt or non-Bt cotton leaf discs; **EE/NF alt.**: *E. kuehniella* eggs were fed alternately with no additional food in 2-day periods, while half of the replicates started with eggs and the other half with no additional food; **NF**: no additional food was provided on the Bt or non-Bt cotton leaf discs. Dishes with *O. majusculus* were incubated at 25 °C, 70% relative humidity and 16 h light. Every second day, leaf discs, cotton pads, and dishes were changed and new food was provided. In the treatments involving spider mites, new spider mites were added on the days in between, because they dried out quickly. In the treatments with alternating food, the food type was switched with every change of the leaf disc (2-day intervals). Replaced leaf discs with *O. majusculus* eggs were further incubated and water was added as needed to keep the cotton pads moist. After 4 days, the number of empty eggs (nymph has hatched = viable egg) and the number of closed eggs (either infertile egg or nymph died before hatching) was counted under a binocular microscope. Males dying within the first 5 days of the experiment were replaced to ensure insemination of females. The experiment was terminated after 24 days. Recorded parameters were survival of males and females (daily), preoviposition period (2-day resolution), percent fertile females, total number of eggs per fertile female (after 24 days), daily fecundity (total number of eggs/number of survived days after the preoviposition period), and hatching rate (number of viable eggs/total number of eggs). The experiment was conducted twice with a total of 10–20 replicates per food treatment (Table 1).

**Table 1:** Treatments included in the experiments with *Orius majusculus* as predators and spider mites, *Spodoptera littoralis* larvae, or *Ephestia kuehniella* eggs as prey. The number of replicates (pairs of *O. majusculus*) at the beginning of the experiments is given. Spider mites and larvae were reared on either non-Bt or Bt cotton. The rearing of *E. kuehniella* did not involve any Bt plants, but the eggs were presented to the predators on either non-Bt or Bt cotton leaf discs

Treatment	Detailed	Replicates (N)	
Abbreviation		Non-Bt	Bt
Experiment with spider mites			
SM	Spider mites	20	20
SM/EE alt	Spider mites and *E. kuehniella* eggs in alternation	20	20
EE	*E. kuehniella* eggs	15	15
EE/NF alt	*E. kuehniella* eggs and no additional food in alternation	20	20
NF	No additional food (leaf disc only)	10	10
Experiment with *S. littoralis* larvae			
SL	*S. littoralis* larvae	20	20
SL/EE alt	*S. littoralis* larvae and *E. kuehniella* eggs in alternation	20	20
SL + EE	*S. littoralis* larvae and *E. kuehniella* eggs simultaneously	20	20
EE	*E. kuehniella* eggs	10	10
EE/NF alt	*E. kuehniella* eggs and no additional food in alternation	20	20
NF	No additional food (lead disc only)	10	10

### Feeding experiments with *S. littoralis* larvae

In a second series of experiments, *S. littoralis* larvae were used as prey instead of spider mites. The experiments followed a similar protocol as described above with the following six food treatments (Table 1): **SL**: prey consisted of live larvae from either Bt or non-Bt cotton, presented on the Bt or non-Bt cotton leaf discs, respectively; **SL/EE alt.**: live larvae from Bt or non-Bt cotton were fed alternately with frozen *E. kuehniella* eggs in 2-day periods, while half of the replicates started with larvae as food and the other half with eggs; **EE**: prey consisted of *E. kuehniella* eggs, presented on Bt or non-Bt cotton leaf discs; **EE/NF alt.**: *E. kuehniella* eggs were fed alternately with no additional food in 2-day periods, while half of the replicates started with eggs and the other half with no additional food; **NF**: no additional food was provided on the Bt or non-Bt cotton leaf discs. **SL + EE**: live larvae from Bt or non-Bt cotton were fed simultaneously with *E. kuehniella* eggs. When leaf discs were changed (every second day), each *O. majusculus* pair received 10 new *S. littoralis* larvae in the respective treatments. Because of high consumption rates in the treatments with exclusive *S. littoralis* prey (**SL**), the number of larvae was increased to 15 from day 8 (first repetition) or day 6 (second repetition) onwards. Prey larvae still alive after the 2-day feeding periods were counted and numbers were subtracted from the number of prey larvae initially provided. In the following, this difference is referred to as “consumed prey”, although other causes of death or escapes might have contributed in addition to killing and consumption by the predators. The trials were terminated after 25 days (1 day after disc change). The experiment was conducted twice with a total of 10–20 replicates per food treatment (Table 1).

### Bt protein analysis

During all experimental repetitions, samples of 7–20 mg fresh weight were collected from Bt and non-Bt cotton leaves (51 each), spider mites (11 Bt, 7 non-Bt), and *S. littoralis* (33 each) for the determination of Mpp51Aa2 using Enzyme-Linked Immunosorbent Assays (ELISA). Because the number of *O. majusculus* adults surviving the treatments with exclusive spider mite or *S. littoralis* feeding after 24 or 25 days was low, additional experiments were set up similar to the main experiments described above, but food treatments consisted exclusively of spider mites or *S. littoralis* larvae from either Bt or non-Bt cotton. Twenty individuals were set up for each food type. After 4 days (spider mites as food) or 5 days (larvae as food), all surviving *O. majusculus* were frozen and pooled to groups of 3–4 individuals. ELISA procedures followed the protocol described previously (Kim et al. 2021; Boss et al. 2023). Tissue was macerated in tris–borate buffer using a bead mill. Samples were diluted according to their expected Bt protein content (Bt cotton leaves 200–1000 × , Bt spider mites 200 × , Bt larvae 100 × , non-Bt spider mites 20 × , and *Orius* and other non-Bt samples undiluted). Ninety-six-well plates were coated with anti-Mpp51Aa2 mouse antibody and a goat anti-Mpp51Aa2 (IgG)-biotin construct served as detection antibody. TMB was added for the colour reaction, which was stopped after 10 min with 6 M phosphoric acid. Absorbance was read at 450 nm and concentrations were determined with an 8-point standard curve derived from purified Mpp51Aa2. Limits of detection were calculated based on 3 × SD of the optical densities (OD) of 4–6 blanks included on each plate.

### Data analysis

Data, available in Online Resource 1, were analysed using R, version 3.6.3. (The R Foundation for Statistical Computing, Vienna, Austria). Survival of adult *O. majusculus* was analysed separately for males and females with the package “survival”. Other parameters were analysed with generalized linear models (GLM). Fixed factors were food type (F), cotton type (C), and their interaction (F × C). For F and C, contrasts were set to orthogonal. Analyses started with models including “experimental repetition” as a random factor, but the factor was removed as it explained no additional variation in most cases. For the distribution and link function used for each parameter, see Tables 2 and 3. Effects of factors and interactions were determined from ANOVA tables with Type III sum of squares (“car” package) with α = 0.05. Significant differences between individual food treatments were determined with Tukey tests (“emmeans” package). For differences in percent fecund females, Games Howell tests (“rstatix” package) were used, because of a lack of variance in some treatments. For survival and for parameters where F × C was significant, F was compared for each C type and C was compared for each F type separately (Tables 2 and 3).

**Table 2:** Life table parameters of *Orius majusculus* adults fed different combinations of spider mites (SM) from Bt or non-Bt cotton, *Ephestia kuehniella* eggs (EE), and no food (NF)

Food treatment	Non-Bt		Bt	Statistic cotton
**Longevity (females) [d]**^a^				**Surfdiff**
SM	20.2 ± 1.21 (20) **B**		14.2 ± 1.46 (20) **b**	*Chi*^*2*^ = *6.3, p* = *0.01*
SM/EE alt	23.2 ± 0.64 (20) **C**		18.9 ± 1.83 (20) **c**	*Chi*^*2*^ = *2.5, p* = *0.1*
EE	21.2 ± 1.85 (15) **BC**		23.6 ± 0.41 (15) **c**	*Chi*^*2*^ = *0.4, p* = *0.5*
EE/NF alt	22.3 ± 1.22 (20) **C**		22.9 ± 0.77 (20) **c**	*Chi*^*2*^ = *0.2, p* = *0.7*
NF	5.7 ± 0.38 (10) **A**		5.1 ± 0.60 (10) **a**	*Chi*^*2*^ = *0.1, p* = *0.7*
*Statistic Food*	*Chi*^*2*^ = *84.2, p* < *0.0001*		*Chi*^*2*^ = *85.9, p* < *0.0001*	
**Longevity (males) [d]**^a^				**Surfdiff**
SM	12.7 ± 1.61 (20) **B**		6.2 ± 0.53 (20) **b**	*Chi*^*2*^ = *13.7, p* = *0.0002*
SM/EE alt	23.3 ± 0.41 (20) **D**		23.3 ± 0.32 (20) **c**	*Chi*^*2*^ = *0.5, p* = *0.5*
EE	20.3 ± 1.78 (15) **CD**		20.3 ± 1.78 (15) **c**	*Chi*^*2*^ = *0.1, p* = *0.7*
EE/NF alt	16.8 ± 1.93 (20) **BC**		18.9 ± 1.80 (20) **c**	*Chi*^*2*^ = *1.3, p* = *0.2*
NF	3.7 ± 0.24 (10) **A**		3.5 ± 0.32 (10) **a**	*Chi*^*2*^ = *0.1, p* = *0.8*
*Statistic Food*	*Chi*^*2*^ = *69.0, p* < *0.0001*		*Chi*^*2*^ = *91.2, p* < *0.0001*	
**Fecund females [%]**				**GLM** (binomial, logit-link)
SM	85 (20)	**ab**	85 (20)	*Chi*^*2*^ = *0, p* = *1*
SM/EE alt	100 (20)	**ab**	80 (20)	
EE	93 (15)	**b**	100 (20)	
EE/NF alt	95 (20)	**b**	100 (20)	
NF	60 (10)	**a**	50 (10)	
*Statistic Food*	*Chi*^*2*^ = *25.9, p* < *0.0001*			*F* × *C: Chi*^*2*^ = *8.4, p* = *0.08*
**Preoviposition time [d]**				**GLM** (gamma, log-link)
SM	1.9 ± 0.25 (17)		2.3 ± 0.38 (17)	*Chi*^*2*^ = *2.4, p* = *0.1*
SM/EE alt	1.5 ± 0.20 (20)		2.0 ± 0.45 (16)	
EE	1.6 ± 0.25 (14)		1.8 ± 0.26 (15)	
EE/NF alt	2.0 ± 0.28 (19)		1.8 ± 0.34 (20)	
NF	1.7 ± 0.42 (6)		2.6 ± 0.40 (5)	
*Statistic Food*	*Chi*^*2*^ = *2.6, p* = *0.6*			*F* × *C: Chi*^*2*^ = *2.0, p* = *0.7*
**Total fecundity [# eggs]**				**GLM** (neg. binomial)
SM	27.7 ± 4.27 (17) **B**		10.5 ± 1.80 (17) **a**	*Chi*^*2*^ = *19.6, p* < *0.0001*
SM/EE alt	115.0 ± 7.81 (20) **C**		78.5 ± 9.58 (16) **b**	*Chi*^*2*^ = *6.7, p* = *0.01*
EE	141.1 ± 14.45 (14) **C**		160.9 ± 13.67 (15) **c**	*Chi*^*2*^ = *0.6, p* = *0.5*
EE/NF alt	96.3 ± 8.58 (19) **C**		100.7 ± 6.30 (20) **b**	*Chi*^*2*^ = *0.1, p* = *0.7*
NF	5.8 ± 1.51 (6) **A**		5.2 ± 1.11 (5) **a**	*Chi*^*2*^ = *0.1, p* = *0.7*
*Statistic Food*	*Chi*^*2*^ = *159.8, p* < *0.0001*		*Chi*^*2*^ = *350.7, p* < *0.0001*	
**Daily fecundity [# eggs]**				**GLM** (gamma, log-link)
SM	1.5 ± 0.17 (17) **A**		0.9 ± 0.10 (17) **a**	*Chi*^*2*^ = *10.3, p* = *0.001*
SM/EE alt	5.2 ± 0.30 (20) **BC**		4.4 ± 0.29 (16) **bc**	*Chi*^*2*^ = *3.4, p* = *0.06*
EE	6.6 ± 0.42 (14) **C**		7.5 ± 0.48 (15) **d**	*Chi*^*2*^ = *2.0, p* = *0.2*
EE/NF alt	4.5 ± 0.35 (19) **B**		4.8 ± 0.25 (20) **c**	*Chi*^*2*^ = *0.6, p* = *0.4*
NF	1.5 ± 0.35 (6) **A**		2.7 ± 1.16 (5) **b**	*Chi*^*2*^ = *1.6, p* = *0.2*
*Statistic Food*	*Chi*^*2*^ = *175.7, p* < *0.0001*		*Chi*^*2*^ = *223.1, p* < *0.0001*	
**Viable eggs [%]**				**GLM** (quasibin., logit-link)
SM	75 (17)		64 (17) **a**	*Chi*^*2*^ = *3.6, p* = *0.06*
SM/EE alt	89 (20)		86 (16) **b**	*Chi*^*2*^ = *3.3, p* = *0.07*
EE	84 (14)		93 (15) **c**	*Chi*^*2*^ = *10.9, p* = *0.001*
EE/NF alt	82 (19)		82 (20) **b**	*Chi*^*2*^ = *2.4, p* = *0.1*
NF	75 (6)		85 (5) **abc**	*Chi*^*2*^ = *0.2, p* = *0.7*
*Statistic Food*	*Chi*^*2*^ = *8.9, p* = *0.06*		*Chi*^*2*^ = *58.4, p* < *0.0001*	

^a^Restricted means ± SE with upper limit = 24 calculated by survfit function of the survival package

Feeding intervals for alternating food (alt.) were 2 days. Statistics for the difference between non-Bt and Bt-cotton (producing Mpp51Aa2) are given in the last column. Statistics for the difference between food treatments are given in the last row of each parameter and significances (p < 0.05) are indicated with letters. When the food × cotton (F × C) interaction was significant, cotton effects were analyzed for each food type and food type effects for each cotton type. Means are given ± SE and the number of replicates in parenthesis (N)

**Table 3:** of *Orius majusculus* adults fed different combinations of *Spodoptera littoralis* larvae (SL) from Bt or non-Bt cotton, *Ephestia kuehniella* eggs (EE), and no food (NF)

Food treatment	Non-Bt		Bt	Statistic cotton
**Longevity (females) [d]**^a^				**Surfdiff**
SL	22.8 ± 0.99 (20) **B**		22.5 ± 1.13 (19) **b**	*Chi*^*2*^ = *0.5, p* = *0.5*
SL/EE alt	25.0 ± 0.00 (20) **B**		24.9 ± 0.15 (20) **bc**	*Chi*^*2*^ = *2.1, p* = *0.2*
SL + EE	24.3 ± 0.44 (20) **B**		25.0 ± 0.00 (20) **c**	*Chi*^*2*^ = *3.2, p* = *0.08*
EE	25.0 ± 0.00 (10) **B**		24.9 ± 0.10 (9) **bc**	*Chi*^*2*^ = *1.1, p* = *0.3*
EE/NF alt	24.3 ± 0.68 (20) **B**		23.1 ± 1.33 (20) **bc**	*Chi*^*2*^ = *1.1, p* = *0.3*
NF	7.2 ± 0.31 (10) **A**		9.1 ± 0.61 (10) **a**	*Chi*^*2*^ = *6.7, p* = *0.01*
*Statistic Food*	*Chi*^*2*^ = *151, p* < *0.0001*		*Chi*^*2*^ = *102, p* < *0.0001*	
**Longevity (males) [d]**^a^				**Surfdiff**
SL	15.4 ± 1.61 (20) **B**		8.7 ± 1.18 (19) **b**	*Chi*^2^ = *10.3*, *p* = *0.01*
SL/EE alt	23.9 ± 1.12 (20) **C**		25.0 ± 0.00 (20) **c**	*Chi*^2^ = *2.0*, *p* = *0.2*
SL + EE	24.0 ± 1.02 (20) **C**		24.6 ± 0.28 (20) **c**	*Chi*^2^ = *1.0*, *p* = *0.3*
EE	24.5 ± 0.47 (10) **C**		22.9 ± 2.0 (9) **c**	*Chi*^2^ = *0.01*, *p* = *0.9*
EE/NF alt	24.4 ± 0.38 (20) **C**		24.4 ± 0.38 (20) **c**	*Chi*^2^ = *0.003*, *p* = *1.0*
NF	4.4 ± 0.21 (10) **A**		4.8 ± 0.24 (10) **a**	*Chi*^2^ = *1.2*, *p* = *0.3*
*Statistic Food*	*Chi*^*2*^ = *115, p* < *0.0001*		*Chi*^*2*^ = *150, p* < *0.0001*	
**Fecund females [%]**				**GLM** (binomial, logit-link)
SL	100 (20)		95 (19)	*Chi*^*2*^ = *0.00, p* = *1.0*
SL/EE alt	95 (20)		100 (20)	
SL + EE	100 (20)		95 (20)	
EE	100 (10)		100 (9)	
EE/NF alt	100 (20)		95 (20)	
NF	80 (10)		90 (10)	
*Statistic Food*	*Chi*^*2*^ = *7.9, p* = *0.2*			*F* × *C: Chi*^*2*^ = *5.6, p* = *0.3*
**Preoviposition time [d]**				**GLM** (gamma, log-link)
SL	3.9 ± 0.66 (20)	**c**	3.2 ± 0.70 (18)	*Chi*^*2*^ = *0.02, p* = *0.9*
SL/EE alt	2.5 ± 0.26 (19)	**bc**	2.5 ± 0.25 (20)	
SL + EE	2.3 ± 0.33 (20)	**bc**	2.9 ± 0.42 (19)	
EE	1.4 ± 0.27 (10)	**a**	1.4 ± 0.29 (9)	
EE/NF alt	1.8 ± 0.27 (20)	**ab**	2.6 ± 0.85 (19)	
NF	1.8 ± 0.37 (8)	**a**	1.2 ± 0.22 (9)	
*Statistic Food*	*Chi*^*2*^ = *43.5, p* < *0.0001*			*F* × *C: Chi*^*2*^ = *7.0, p* = *0.2*
**Total fecundity [# eggs]**				**GLM** (neg. binomial)
SL	38.5 ± 3.99 (20)	**b**	33.6 ± 4.91 (18)	*Chi*^*2*^ = *0.00, p* = *0.7*
SL/EE alt	118.7 ± 3.87 (19)	**c**	107.9 ± 7.41 (20)	
SL + EE	140.9 ± 6.54 (20)	**cd**	124.7 ± 10.27 (19)	
EE	170.7 ± 27.34 (10)	**d**	185.4 ± 13.22 (9)	
EE/NF alt	117.2 ± 8.74 (20)	**c**	106.2 ± 12.70 (19)	
NF	5.6 ± 1.43 (8)	**a**	9.9 ± 2.02 (9)	
*Statistic Food*	*Chi*^*2*^ = *410, p* < *0.0001*			*F* × *C: Chi*^*2*^ = *6.0, p* = *0.4*
**Daily fecundity [# eggs]**				**GLM** (gamma, log-link)
SL	2.1 ± 0.18 (20)	**b**	1.7 ± 0.21 (18)	*Chi*^*2*^ = *0.00, p* = *0.5*
SL/EE alt	5.3 ± 0.17 (19)	**c**	4.8 ± 0.32 (20)	
SL + EE	6.4 ± 0.23 (20)	**cd**	5.6 ± 0.44 (19)	
EE	7.2 ± 3.56 (10)	**d**	7.9 ± 0.52 (9)	
EE/NF alt	5.1 ± 0.32 (20)	**c**	5.0 ± 0.46 (19)	
NF	1.0 ± 0.19 (8)	**a**	1.2 ± 0.20 (9)	
*Statistic Food*	*Chi*^*2*^ = *423, p* < *0.0001*			*F* × *C: Chi*^*2*^ = *4.0, p* = *0.5*
**Viable eggs [%]**				**GLM** (quasibin., logit-link)
SL	79 (20)	**a**	68 (18)	*Chi*^*2*^ = *0.4, p* = *0.5*
SL/EE alt	81 (19)	**ab**	81 (20)	
SL + EE	86 (20)	**b**	85 (19)	
EE	85 (10)	**b**	88 (9)	
EE/NF alt	76 (20)	**a**	81 (18)	
NF	74 (8)	**ab**	74 (9)	
*Statistic Food*	*Chi*^*2*^ = *28.1, p* < *0.0001*			*F* × *C: Chi*^*2*^ = *3.9, p* = *0.6*
**Consumed prey after 2d [# larvae]**				**GLM** (gamma, log-link)
SL	9.3 ± 0.49 (20)	**c**	9.0 ± 0.50 (19)	*Chi*^*2*^ = *1.6, p* = *0.2*
SL/EE alt	2.4 ± 0.28 (20)	**b**	3.4 ± 0.25 (20)	
SL + EE	1.1 ± 0.11 (20)	**a**	1.1 ± 0.15 (20)	
*Statistic Food*	*Chi*^*2*^ = *518.4, p* < *0.0001*			*F* × *C: Chi*^*2*^ = *5.4, p* = *0.1*

^a^Restricted means ± SE with upper limit = 25 calculated by survfit function of the survival package

In the SL + EE treatment, both prey types were offered simultaneously. Feeding intervals for alternating food (alt.) were 2 days. Statistics for the difference between non-Bt and Bt-cotton (producing Mpp51Aa2) are given in the last column. Statistics for the difference between food treatments are given in the last row of each parameter and significances (p < 0.05) are indicated with letters. When the food × cotton (F × C) interaction was significant, cotton effects were analyzed for each food type and food type effects for each cotton type. Means are given ± SE and the number of replicates in parenthesis (N)

## Results

### Feeding experiments with spider mites

**Fig. 1:**
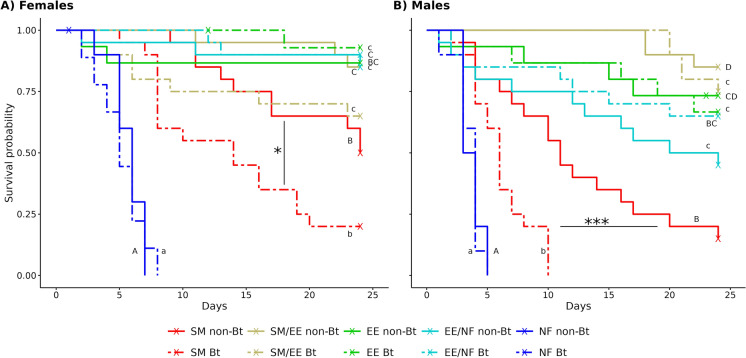
Survival of *Orius majusculus* females (**A**) and males (**B**) raised on different food types: *Tetranychus urticae* spider mites (SM), *Ephestia kuehniella* eggs (EE), no additional food (NF), or two food types fed in alternation (SM/EE or EE/NF). Spider mites were reared either on Mpp51Aa2-producing cotton (Bt) or near-isogenic cotton (non-Bt). All prey types were presented on the respective Bt or non-Bt cotton leaf discs. Different letters indicate significant differences among food types (capital letters for non-Bt, small letters for Bt treatments). Asterisks indicate significant differences between cotton types (*p < 0.05, ***p < 0.001)

Longevity of *O. majusculus* females fed with spider mites reared on Bt cotton was 30% lower (14 days) than longevity of females fed with non-Bt mites (20 days) (*p* = 0.01, Table 2, Fig. 1A). By the end of the experiments (24 days), 4 of 20 females (20%) in the Bt cotton treatment and 10 of 20 females (50%) in the non-Bt cotton treatment were alive. When spider mites and *E. kuehniella* eggs were fed in alternation, 13 of 20 females (65%) survived the experimental period in the Bt and 17 of 20 females (85%) in the non-Bt group (*p* = 0.1). The different food treatments had a high impact on longevity (*p* < 0.0001). Without additional food (leaf disc only), *O. majusculus* females died on average after 5.7 days. Females fed with spider mites lived significantly longer than those without additional food. Females fed with *E. kuehniella* eggs, either exclusively or in alternation with spider mites or no additional food lived longest (Table 2). Male longevity showed a similar pattern (Table 2, Fig. 1B). Males fed spider mites from Bt lived half as long (6.2 days) as males fed spider mites from non-Bt cotton (12.7 days) (*p* = 0.0002). The different food types had a strong impact on male longevity (*p* < 0.0001).

The percentage of fecund females was not influenced by cotton type (*p* = 1.0). Less fertile females were observed when only the leaf disc without additional food was provided to the predators compared with either *E. kuehniella* eggs as exclusive prey and eggs and no additional food in alternation (Table 2). No difference among cotton and food types was observed for the preoviposition time, which was on average 1.9–2.3 days (*p* ≥ 0.1, Table 2).

**Fig. 2:**
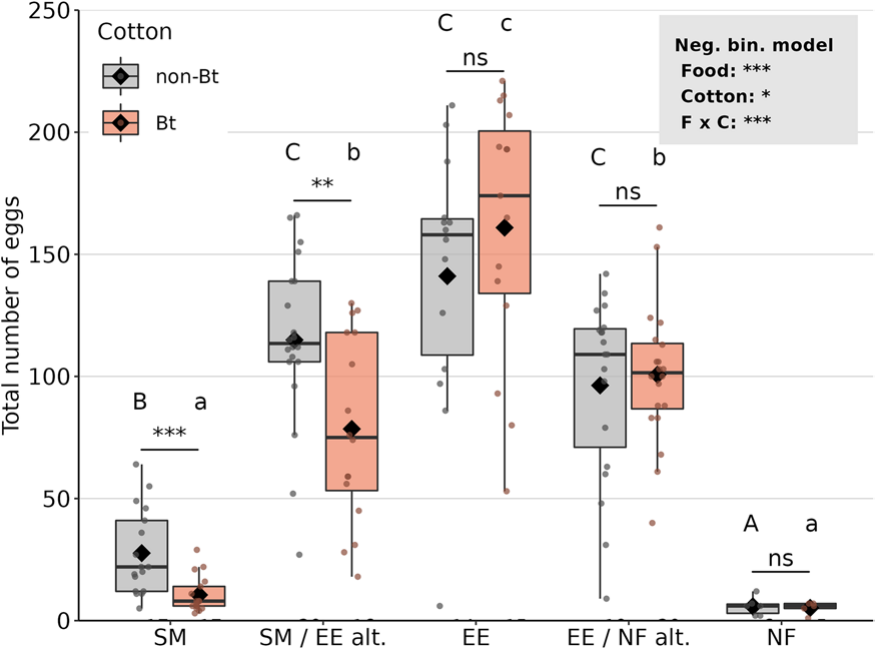
Total fecundity of *Orius majusculus* raised on different food types: *Tetranychus urticae* spider mites (SM), *Ephestia kuehniella* eggs (EE), no additional food (NF), or two food types fed in alternation (SM/EE alt. or EE/NF alt.). Spider mites were reared either on Mpp51Aa2-producing cotton (Bt) or near-isogenic cotton (non-Bt). All prey types were presented on the respective Bt or non-Bt cotton leaf discs. Dots represent individual values (N = 5–20), black rhombuses means, black horizontal lines medians, hinges 25th and 75th percentiles, and whiskers the smallest or largest values no further than 1.5 × IQR from the hinges. Results of GLM with fixed factors food (F) and cotton type (C) are presented in the grey box. Letters display sigificant differences between prey types, analysed separately for the two cotton types. Asterisks indicate significant differences between cotton types (*p < 0.05, **p < 0.01, ***p < 0.001)

Total fecundity was less than half when *O. majusculus* females were fed with spider mites from Bt (10.5 eggs in total) compared with non-Bt cotton (27.7) (*p* < 0.0001, Fig. 2, Table 2). Similarly, significantly less eggs were laid when spider mites from Bt cotton and *E. kuehniella* eggs were fed in alternation (78.5) compared with the non-Bt treatment (115.0) (*p* = 0.01). Without additional food, females managed to lay on average 5.5 eggs, significantly less than when fed with spider mites. Compared with the spider mite treatments, significantly more eggs were produced when *E. kuehniella* eggs were provided, either exclusively or in alternation with spider mites or no additional food (Fig. 2, Table 2). A similar pattern was observed for daily fecundity. While spider mites as exclusive food resulted in fewer eggs in the Bt compared with the non-Bt treatment (*p* = 0.001), a comparable effect was borderline non-significant when spider mites and *E. kuehniella* eggs were fed in alternation (*p* = 0.06).

The percentage of viable eggs (hatching rate) laid by *O. majusculus* females was not affected by cotton type in the treatments involving spider mites (*p* ≥ 0.06). However, the hatching rate of eggs from females fed with spider mites from Bt cotton was lower than the rate from females fed with *E. kuehniella* eggs, either exclusively or in alternation with spider mites or no additional food (Table 2).

For all parameters, no differences between the Bt and the non-Bt treatment were observed when no spider mites were involved as a vehicle for Mpp51Aa2 transfer. In those treatments, only the leaf disc contained the Bt protein, while *E. kuehniella* eggs, containing no Bt protein, were the same in both treatments. One exception was the hatching rate in exclusive *E. kuehniella* treatments, which was higher in the Bt compared with the non-Bt treatment (*p* = 0.001, Table 2).

### Feeding experiments with *S. littoralis* larvae

**Fig. 3:**
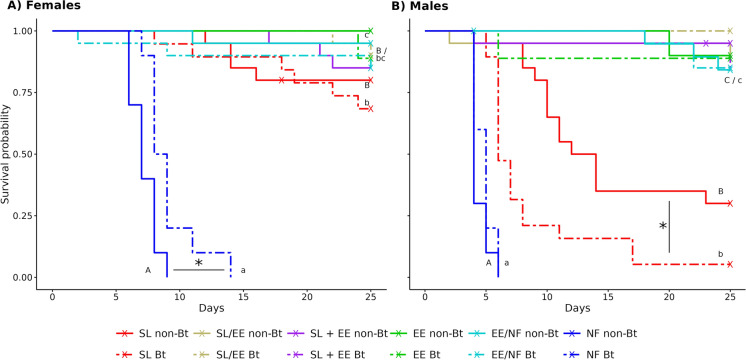
Survival of *Orius majusculus* females (**A**) and males (**B**) raised on different food types: *Spodoptera littoralis* larvae (SL), *Ephestia kuehniella* eggs (EE), no additional food (NF), two food types fed in alternation (SL/EE or EE/NF) or simultaneously (SL + EE). Larvae were fed with Mpp51Aa2-producing cotton (Bt) or near-isogenic cotton (non-Bt). All prey types were presented on the respective Bt or non-Bt cotton leaf discs. Different letters indicate significantly different food types (capital letters for non-Bt, small letters for Bt treatments). Asterisks indicate significant differences between cotton types (*p < 0.05)

In the experiments with *S. littoralis* larvae as prey, female longevity showed no significant differences between Bt and non-Bt treatments, except in the treatments without additional food, where females kept on Bt leaf discs lived 9.1 days and those on non-Bt leaf discs 7.2 days (Fig. 3A, Table 3). Females provided with food lived longer than in the treatments without additional food and females fed with *S. littoralis* larvae from Bt cotton exclusively had a higher mortality than females fed with Bt larvae and *E. kuehniella* eggs simultaneously (*p* < 0.05). Other food treatments had no impact on female longevity (Table 3, *p* ≥ 0.05). *Orius majusculus* males fed with *S. littoralis* larvae reared on Bt cotton survived half as long (8.7 days) as males fed with non-Bt larvae (15.4 days) (*p* = 0.01, Fig. 3B, Table 3). After 25 days, 1 of 19 males in the Bt cotton treatment and 6 of 20 males in the non-Bt cotton treatment were alive. In all treatments where *E. kuehniella* eggs were provided as food, more than 75% of the males survived the experimental period, regardless if Bt or non-Bt treatment. The different food types had a high impact on male longevity (*p* < 0.0001). Without additional food, *O. majusculus* males died on average after 4.4–4.8 days. Males fed with *S. littoralis* larvae lived significantly longer than those without additional food. Males fed with *E. kuehniella* eggs, either exclusively or in alternation with larvae or in alternation with no additional food, or simultaneously with larvae, lived longest (Fig. 3B, Table 3).

The percentage of fecund females was not influenced by cotton type (*p* = 1.0) or food type (*p* = 0.2) (Table 3). While preoviposition time also was similar among cotton types (*p* = 0.9), females without additional food and females fed with *E. kuehniella* eggs exclusively produced first eggs earlier than females fed with *S. littoralis* larvae (exclusively or in alternation or simultaneously with *E. kuehniella* eggs). Longest preoviposition periods were observed for females fed with larvae exclusively.

**Fig. 4:**
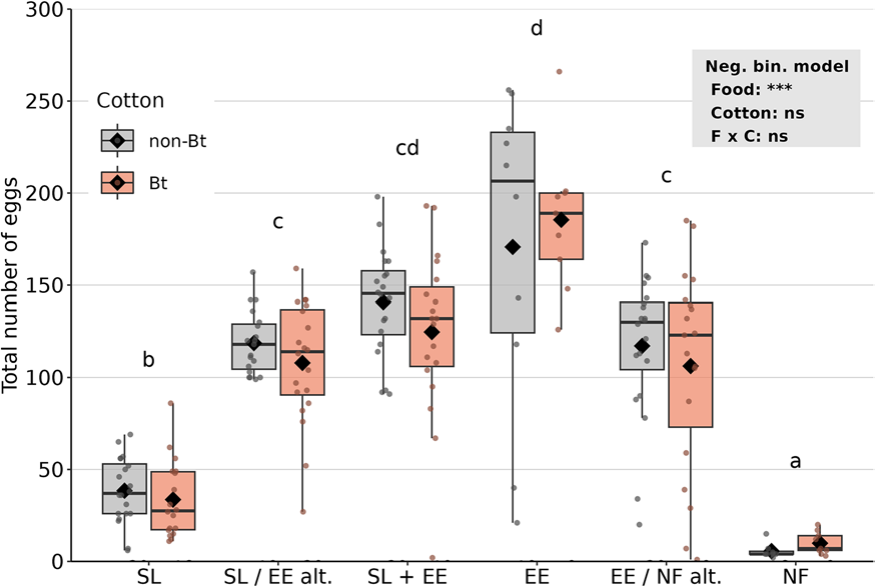
Total fecundity of *Orius majusculus* raised on different food types: *Spodoptera littoralis* larvae (SL), *Ephestia kuehniella* eggs (EE), no additional food (NF), two food types fed in alternation (SL/EE alt. or EE/NF alt.), or simultaneously (SL + EE). Larvae were reared either on Mpp51Aa2-producing cotton (Bt) or near-isogenic cotton (non-Bt). All prey types were presented on the respective Bt or non-Bt cotton leaf discs. Dots represent individual values (N = 8–20), black rhombuses means, black horizontal lines medians, hinges 25th and 75th percentiles, and whiskers the smallest or largest values no further than 1.5 × IQR from the hinges. Results of GLM with fixed factors food (F) and cotton type (C) are presented in the grey box (***p < 0.001). Letters display sigificant differences between prey types

Total fecundity was not affected by cotton type (*p* = 0.7, Fig. 4, Table 3), but food treatment had a strong effect (*p* < 0.0001). Without additional food, female *O. majusculus* managed to lay on average 5.6 eggs (non-Bt leaf discs) and 9.9 eggs (Bt leaf discs). More eggs were produced when females were provided *S. littoralis* larvae exclusively (38.5/33.6) and even more eggs when *E. kuehniella* eggs were fed, either in alternation, simultaneously, or exclusively (≥ 106). The highest fecundity was observed in the treatments with exclusive *E. kuehniella* food (170/185 eggs) (Fig. 4, Table 3). The same pattern was observed for daily fecundity (Table 3).

The hatching rate of the eggs laid by *O. majusculus* females was not affected by cotton type (*p* = 0.5), while prey type was significant (*p* < 0.0001) (Table 3). The percentage of viable eggs was lower when the predators were fed *S. littoralis* larvae exclusively or when *E. kuehniella* eggs were alternated with no additional food, compared with the treatments with *E. kuehniella* eggs only or larvae + eggs simultaneously.

When analyzing the number of consumed prey larvae, a clear food-treatment effect was observed (*p* < 0.0001), while cotton type had no effect (*p* = 0.2). An average of 9.0–9.3 larvae was consumed (defined as not retrieved) per 2-day feeding period when larvae were the only available prey. In contrast, when larvae and *E. kuehniella* eggs were provided to the predators alternately, only 2.4 (Bt) or 3.4 (non-Bt) larvae were consumed. When eggs were available at the same time as larvae, approximately 1.1 larva was consumed after the 2-day feeding periods.

### Mpp51Aa2 concentrations in leaves, prey and predators

Bt cotton leaves contained a median of 110 µg Mpp51Aa2/g fresh weight in the experiments with spider mites and 139 µg/g in the experiments with *S. littoralis* larvae (Table 4). Prey species contained 6–10 times less Bt protein compared to cotton leaves, i.e., spider mites 19.7 µg/g and larvae 15.0 µg/g. In *O. majusculus* feeding 4–5 days on Bt protein-containing prey, 100–500 times less Mpp51Aa2 was measured compared with their prey: 0.034 µg/g when fed with spider mites and 0.15 µg/g when fed with larvae (Table 4).

**Table 4:** Median Mpp51Aa2 (previously mCry51Aa2) concentrations (in µg/g FW) of cotton leaves, spider mites collected from cotton, *Spodoptera littoralis* larvae fed overnight on cotton, and *Orius majusculus* adults feeding for 4 days on mites or 5 days on larvae

	Non-Bt		Bt	
	Mpp51Aa2	n	Mpp51Aa2	n
Spider mites as prey				
Leaves	0.016 (0.0021; 0.94)	23	109.5 (72.2; 159.1)	23
Spider mites	0.023 (0; 0.36)	7	19.7 (14.1; 31.4)	11
*O. majusculus*	0.0012 (0; 0.011)	11	0.034 (0.0078; 0.16)	8
Larvae as prey				
Leaves	0 (0; 0.0034)	28	138.6 (94.8; 173.6)	28
Larvae	0 (0; 0)	33	15.0 (9.3; 28.3)	30
*O. majusculus*	0 (0; 0.0032)	10	0.15 (0.061; 0.46)	10

Minimum and maximum values are given in parenthesis. n is the number of analysed samples. Bt indicates genetically engineered cotton producing Mpp51Aa2 (MON 88702), non-Bt indicates the non-transformed near-isogenic line (DP393)

## Discussion

### Effects of Bt cotton on *O. majusculus* performance

When exclusively fed with frozen spider mites from Bt cotton, *O. majusculus* males and females showed reduced longevity and females produced fewer eggs (total fecundity and daily fecundity) compared with bugs fed with spider mites from non-Bt cotton. This confirms findings by Kim et al. (2021), who also reported reduced longevity and fecundity when *O. majusculus* females were fed with living spider mites from Mpp51Aa2-producing cotton. Because Kim et al. (2021) started their experiments with 5-day-old nymphs, their observed effects might have resulted from effects in the nymphal stage (leading to lower adult weight) in combination with effects during the adult feeding period. The current experiments, however, show clearly that Bt spider mites caused effects on male and female *O. majusculus* when test specimens had the same history in their developmental period until adulthood.

When *O. majusculus* adults were fed exclusively with *S. littoralis* larvae that have fed on Bt cotton, effects were only observed on male longevity, but not on female longevity or fecundity. Males suffered higher mortality compared with females in treatments without food or with food of suboptimal nutritional value, such as spider mites or *S. littoralis* larvae. This higher sensitivity of males to food quality also might have resulted in higher sensitivity towards the Bt toxin compared with females. *Spodoptera littoralis* larvae contained 25% less Mpp51Aa2 than spider mites. This rather small difference might be responsible for the observed difference in effects between prey types. However, it is likely that other factors, such as differences in metabolism that might have influenced the bioactivity of the measured Mpp51Aa2 in spider mites and larvae, as well as differences in the general suitability of the prey species for the predator might also have impacted the toxicity.

Similar to the current study on adults, Boss et al. (2023) reported no Bt effect when *S. littoralis* larvae were used as food for *O. majusculus* nymphs during their developmental period, but strong effects when spider mites were used. It has to be noted, however, that ELISA measurements suggested a larger difference in Mpp51Aa2 concentrations between spider mites (30–103 µg/g fresh weight) and larvae (18 µg/g) in the study by Boss et al. (2023) compared with the present study.

The key question of the current study was if supplementing a spider mite or *S. littoralis* diet with high quality food that does not contain Bt protein (*E. kuehniella* eggs) could mitigate Bt effects. This was realized by feeding *O. majusculus* with spider mites or larvae and *E. kuehniella* eggs in alternation using 2-day intervals. Predator longevity and fecundity was much better than when fed with spider mites or larvae alone. However, a significant Bt effect on total fecundity was still visible with spider mites as prey, although less pronounced than for the spider mite-only treatments. Once more, a similar effect was reported by Boss et al. (2023) on developmental parameters: feeding alternately mites and eggs reduced Bt effects on development time and adult weight, but did not completely compensate them (Boss et al. 2023). In the assay with *S. littoralis* as prey, the effect on male longevity was no longer observed when prey larvae were supplemented with *E. kuehniella* eggs and no Bt effects were evident when both prey types were provided simultaneously.

### Effects of different food types on *O. majusculus* performance

This study confirms earlier findings that mites are of lower nutritional value than *E. kuehniella* eggs for *O. majusculus* (Boss et al. 2023; Kim et al. 2021) and other *Orius* species (Bonte et al. 2017). Nevertheless, the study shows that longevity and fecundity with mites were better than without additional food. This demonstrates that *O. majusculus* consumed the frozen spider mites in our experiment. Performance of adult bugs fed with *S. littoralis* larvae generally was better than that of *O. majusculus* fed with spider mites, although the current study does not allow a direct statistical comparison, because the two prey types were not fed in the same experiments. The numbers of consumed larvae clearly show that *O. majusculus* preyed readily on *S. littoralis* when no other food was available. *Ephestia kuehniella* eggs was the best food for predator survival and reproduction, also confirming previous results for this (Boss et al. 2023) and other *Orius* species (Bonte et al. 2017). Interestingly, when eggs and no additional food was supplied in alternation, performance of *O. majusculus* was only impaired to some degree and similar to the performance when eggs and mites or eggs and larvae were provided in alternation. In addition, the number of consumed larvae was low when fed in alternation with eggs and even lower when fed simultaneously with eggs. This shows that *O. majusculus* prefers more suitable (“higher quality”) food when available. Furthermore, the predator can cope well with 2-day gaps without additional food or without high quality food and consumes a larger quantity of lower quality food only when no high-quality food is available for longer time periods.

In the treatments with simultaneous availability of eggs and larvae, fecundity of *O. majusculus* females was somewhat lower (albeit not significant) than in the treatments where eggs were provided exclusively, which might be due to the fact that *O. majusculus* females might have been disturbed by the larvae. Also, preoviposition time was longer in treatments with larvae compared with treatments without larvae.

### Suitability of frozen prey

Frozen spider mites dried out quickly, which likely reduced the ability or preference of *O. majusculus* to consume them. Thus, nutritional suitability of frozen mites might be considered lower than of living mites. To minimize this effect, new spider mites were provided daily. Compared with Kim et al. (2021) who fed live spider mites to *O. majusculus* until death (up to 5 weeks), daily fecundity in the current experiment was similar in the Bt treatment (0.9 eggs per day), but half in the non-Bt treatment (current study 1.47 eggs per day, Kim et al. 2.9 eggs per day). Nevertheless, frozen spider mites increased longevity and total fecundity of *O. majusculus* 2–4 times compared with no additional food. For *E. kuehniella* eggs, freezing is less of a problem as the egg shell remains intact and prevents drying out.

For the second set of experiments, living *S. littoralis* larvae were used because they were relatively easy to produce in suitable quantities. It was shown, however, that frozen *Spodoptera* sp. larvae also were suitable food for *Orius* sp. (Ren et al. 2022). Whenever necessary, freezing is thus a good option to store and ship large amounts of prey without the need for constant rearing infrastructure. However, suitability of frozen prey depends on prey type (e.g., eggs or larvae) and on the ability of the predator to handle and digest dead prey. In addition, Bt proteins are likely to deteriorate quicker in frozen and thawed prey items compared with living prey that can continuously feed on Bt plant leaf discs, which might have also affected the results of the current study.

### Implications

Adverse effects of prey with low nutritional quality and high Mpp51Aa2 concentrations, such as spider mites, were evident for *O. majusculus* development (Boss et al. 2023), longevity, and reproduction (Kim et al. 2021 and this study) when representing the sole food source. Spider mites have been reported to contain the highest measured Bt protein concentrations among arthropods in Bt crops (Romeis et al. 2019; Torres and Ruberson 2008) and can thus be considered a worst-case exposure scenario for predators. *Orius* spp. have been reported to also feed on tissue of certain plants (Armer et al. 1998; Pumariño and Alomar 2012), which might expose them to even higher concentrations, if the respective plant produces insecticidal proteins. In the current leaf disc setup with cotton, however, the presence of plant material did not improve longevity compared with a treatment without leaf disc, suggesting that plant feeding in cotton is limited (Boss et al. 2023).

Alternative food can partly or fully mitigate Bt effects, depending on when and how long it is available. *Orius* spp. are known to consume a variety of soft-bodied insects in the field as well as pollen (Ballal and Yamada 2016; Corey et al. 1998; Lattin 1999). Whether or not the food scenarios deployed by Boss et al (2023) and in this study are realistic is difficult to judge. In the field, however, it is likely that a diverse mixture of prey species is available and that also the Bt protein concentrations of prey items range from zero to high (Eisenring et al. 2017). In particular phloem-feeding species, like aphids, whiteflies, and some leafhoppers contain no or only trace amounts of Bt protein (Eisenring et al. 2017; Meissle and Romeis 2018; Boss et al. 2023), as do non-feeding stages, such as insect eggs or pupae (Meissle et al. 2021). Furthermore, Mpp51Aa2 at doses produced in Bt cotton and observed in herbivorous prey species, resulted in significant, but limited effects on *O. majusculus* development, longevity and fecundity only in worst case exposure scenarios with spider mites (Kim et al. 2021; Boss et al. 2023, and this study). Choice experiments indicated, however, that spider mites are not a preferred prey of *O. majusculus* (Boss et al. 2023) and *O. sauteri* (Xu and Enkegaard 2009). When *S. littoralis* larvae were provided to *O. majusculus* as prey, no effects on female longevity and reproduction were evident despite relatively high Bt protein concentrations in the larvae (Boss et al. 2023 and this study). No effects were observed when spider mites and alternative prey were available simultaneously (Boss et al. 2023 and this study). The results of this study thus confirm the outcome of the environmental risk assessment (summarized in USDA-APHIS 2020) that predatory bug populations are unlikely to suffer harm from MON 88702 cotton in the field, because it is unlikely that their diet consists predominantly of spider mites and other prey types with similarly high Bt protein concentrations. In fact, arthropod communities have been studied in field experiments with MON 88702 cotton over three years at 5–6 sites in various cotton growing regions of the USA (Asiimwe et al. 2023). No significant differences in abundance of *Orius* spp. and other predatory Heteroptera in MON 88702 compared to unsprayed conventional cotton (DP393) were observed. As there is no evidence that biological control by predatory bugs is disrupted, MON 88702 cotton may thus be a valuable tool for the management of sucking pests in integrated production systems. Efficient control of target pests by MON 88702 in combination with functional populations of natural enemies may also contribute to a reduced need for pesticide applications, which is an important goal of integrated pest management.

### Supplementary Information

Below is the link to the electronic supplementary material.

Supplementary file1 (XLSX 96 KB)

## Data Availability

All data used for this manuscript are available in the electronic supplementary material.
